# *Campylobacter fetus*-induced primary psoas abscess in patient with gouty arthritis: A case report and literature review

**DOI:** 10.1097/MD.0000000000036333

**Published:** 2023-12-22

**Authors:** Xiaodong Luo, Yanfang He, Daogang Zha, Chunyu Kang, Yuan Sijie

**Affiliations:** a Department of General Practice, Nanfang Hospital, Southern Medical University, Guangzhou, China.

**Keywords:** *Campylobacter fetus*, case report, eosinophils, meropenem, psoas abscess

## Abstract

**Rationale:**

*Campylobacter fetus* is rare pathogen with high mortality rate in immunosuppressive hosts. This study aimed to summarize clinical and pathological presentation of *C fetus* induced psoas abscess.

**Patient concerns:**

A 66-year-old male patient with long medical history of poorly-controlled gouty arthritis and steroid intake complained of a severe low back pain. Physical examination showed tenderness in his psoas.

**Diagnoses:**

The patient underwent puncture biopsy to the lesion in the psoas under ultrasound guidance. The lesion was indicated as abscess by pathological examination, and its pathogen was indicated as *C fetus* by the next generation sequencing.

**Interventions:**

Meropenem 1 g q8.h were administered intravenously for 10 days. Then the antibiotic treatment was switched to amoxicillin/clavulanate potassium 0.375g q.8.h and levofloxacin 0.5g q.d oral administration when discharge.

**Outcomes:**

The patient’s fever and low back pain improved and infectious parameters declined. He was discharged in good general condition with advice for further monitoring and therapy. In the first month follow-up, the patient did not report recurrence or aggravation of his symptoms.

**Lessons:**

*C fetus* should be noticed in immunosuppressive patient with exposure to livestock who present with rare systematic or local invasive infection. We advocated the meropenem for the first-line treatment against *C fetus*.

## 1. Introduction

Psoas abscess is an infection-invasive condition rarely reported worldwide.^[1]^ It is traditionally classified into primary and secondary according to its origin and spreading distance. Individuals with immunosuppression or underlying diseases are susceptible to developing psoas abscess. The common pathogens are *Staphylococcus aureus, Escherichia coli, Bacteroides species*, and *Mycobacterium tuberculosis*.^[2]^ Besides, some rare pathogens may also induce the psoas abscess, including *Campylobacter fetus*. Till recent, only 2 cases reported *C fetus*-induced secondary psoas abscess associating with spondylitis,^[3,4]^ while no primary case is reported. Although the clinical outcomes were favorable, it may become fatal if invasive *C fetus* infection could not be diagnosed and treated in time. In the past decades, *C fetus* infection was difficult to diagnose due to low positive culture, and the number of cases was probably underestimated.^[5]^ Owning to the rare cases, the development in early diagnosis and treatment of *C fetus*-induced secondary psoas abscess is limited. Herein, we reported a case of *C fetus*-induced primary psoas abscess in a Chinese rural male with long medical history of gouty arthritis and oral steroids intake. Blood cultures did not grow any pathogenic bacteria. He was treated initially with a combination of ceftriaxone and levofloxacin then meropenem along for 2 weeks. The clinical response was favorable, and the inflammatory parameters significantly declined when the patient was discharged. The present case report provides experiments in diagnosis and treatment toward primary *C fetus* infection in psoas.

## 2. Case report

**Figure 1.**
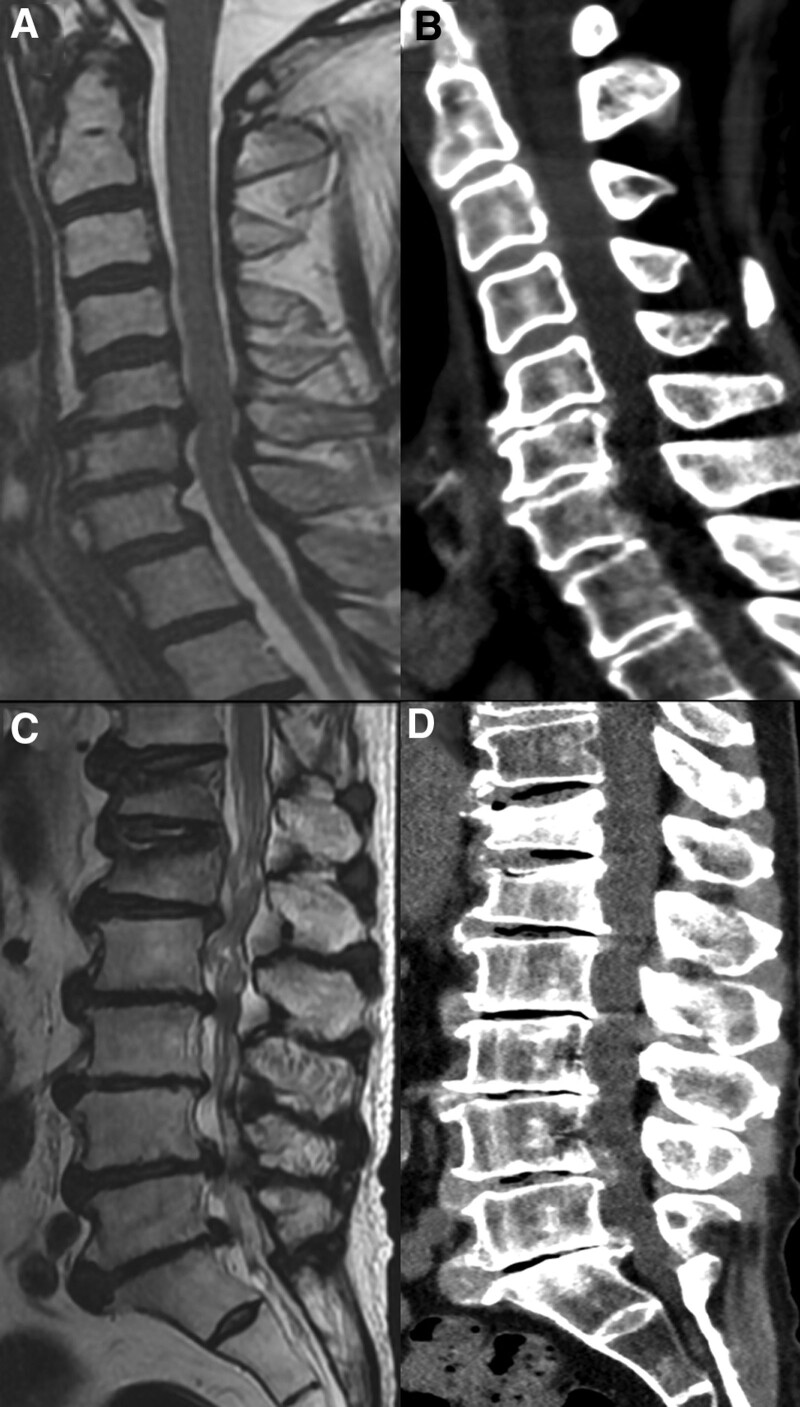
Imaging examination of cervical and lumbar vertebras. (A) MRI of cervical in sagittal view. (B) CT of cervical in sagittal view. (C) MRI of lumbar in sagittal view. (D) CT of cervical in sagittal view. CT = computerized tomography scan, MRI = magnetic resonance imaging.

A 66-year-old Chinese rural male with a past medical history of gouty arthritis presented to the spine surgery department of Nanfang Hospital, Southern Medical University for paralysis in left upper and lower limbs. The patient reported it started approximately 1 year ago. He endorsed associated symptoms of worsening neck and low back pain for the past 1 month. On initial presentation, the patient’s blood pressure, heart rate and temperature were normal. The spinal physical examination revealed limitation in neck and waist motion, positive Hoffmann symptoms, tenderness in several lumbar intervertebral discs, and amyotrophy in 2 lower limbs. Magnetic resonance imaging (MRI) and Computerized tomography scan revealed multiple cervical and lumbar intervertebral discs herniation combined with secondary stenosis, and fresh vertebral compression fractures in T12 and L1 (Fig. 1A–D). Given the urgent operation indications in cervical spinal, the orthopedists planned to perform cervical decompression next day. However, initial laboratory exam reported mild leukocytosis of 10.00 × 10^9^/L combined with elevated C-reactive protein (CRP) of 59.29mg/L and ProCT of 1.040 ng/mL, hinting potential infectious risks. The orthopedists had to postpone the operation and transmit the patient to the department of general practice.

**Figure 2.**
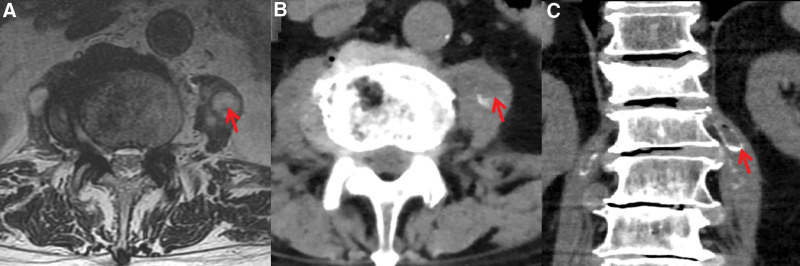
Imaging examination of lumbar vertebras. (A) MRI of cervical in horizontal view. (B) CT of cervical in horizontal view. (C) CT of cervical in coronal view. Red arrows indicated the psoas lesions. CT = computerized tomography scan, MRI = magnetic resonance imaging.

**Figure 3.**
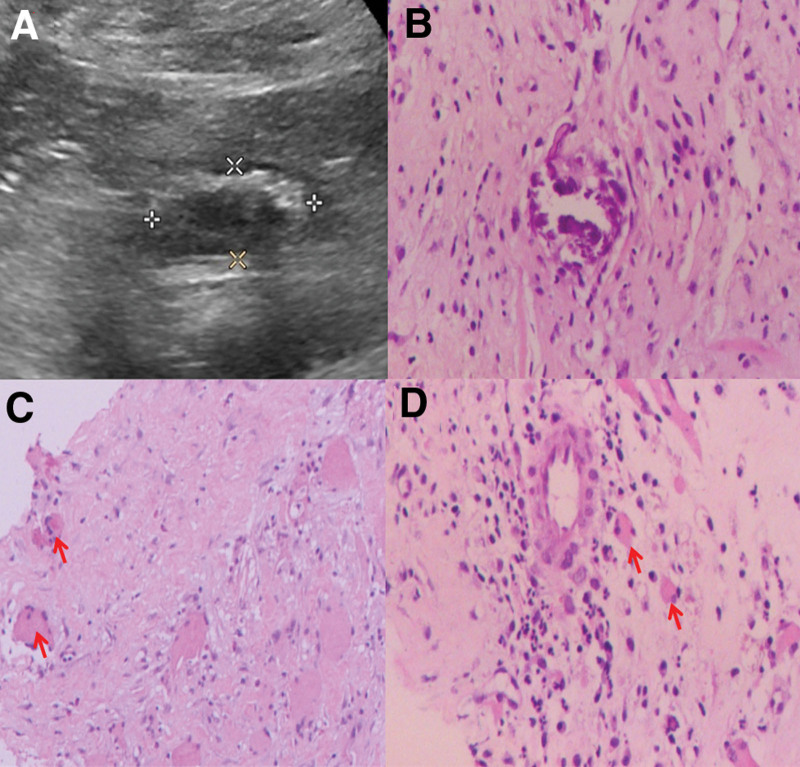
Biopsy of psoas lesion. (A) Ultrasound image of lesion for puncture biopsy. (B–D) Pathological examination of psoas lesion (1000×). Red arrows indicated the eosinophils.

After admission, the patient complained of suffering from a severe low back pain, enforcing him bedridden, and to require tramadol 0.1 g q.d i.m. to resolve. He also reported the acute gouty arthritis usually relapsed in the past 3 years, during which he was prescribed plenty of metacortandracin as analgetic. The general physical examination revealed thickening of fat around the face (moon face), telangiectasias, muscle wasting and weakness in 4 limbs, thinning of the skin and slight violet rashes on abdomen. Besides, a peculiar tenderness on the left psoas, which could not be well explained by the lumbar intervertebral discs herniation or compressive fracture, caught our attention. As the cortisol and adrenocorticotropic were normal, the metacortandracin was stopped immediately. According to the infectious disease consultation, the patient was initialed on empiric intravenous (i.v.) antibiotic treatment of ceftriaxone sodium 2g q.d. Two days after, the mycoplasma pneumoniae condensing set test reported positive result of 1:160, and oral azithromycin 0.5g q.d administration was added into treatment. The patient reported emerging anaphylactic rashes on his chest, and the antibiotic treatment was stopped in case of aggravated allergies. Owning to the complicated condition, the hospitalized disease symposium was performed. The suspected lesions in the psoas were found in the lumbar MRI and computerized tomography (Fig. 2A and B). The puncture biopsy guided by ultrasound was performed to yield the tissue for pathogen next generation sequencing (NGS) (RNA and DNA), and pathological examination (Fig. 3A). In the total 129,414,630 sequences of RNA and 121,331,807 sequences of DNA, the NGS reported 177 RNA sequences with 0.52% abundance and 426 DNA sequences with 11.03% abundance, which were all belonged to the bacteria, *C fetus*. Subsequently, the pathological examination reported chronic abscess associated with lytic necrosis, fibroplasia, calcification and eosinophilia infiltration in the lesion (Fig. 3B–D). Immediately, the antibiotic treatment was escalated to ceftriaxone sodium 2 g q.d and levofloxacin 0.5 g q.d intravenous administration. During the treatment, the patient appeared fever associated with arthritis in his left ankle and knee joints, which he reported was similar to the pervious acute gouty arthritis. Emergent tests of complete blood count, CRP, Procalcitonin (PCT), uric acid and 2 sets of blood culture were performed, and results reported the leukocyte, CRP, and PCT elevated to 15.00 × 10^9^/L, 228.42 mg/mL, and 0.864 ng/mL, while the uric acid remained normal as previous (<420 mmol/L). The fever and arthritis were ameliorated after oral ibuprofen and colchicine administrations. In consideration of antibiotic resistance, the antibiotic treatment was escalated to meropenem 1g i.v. q.8.h. The oral ibuprofen and colchicine administrations were performed when needed. After 10-day treatment, patient reported the fever, severe low back pain and psoas tenderness significant improved, and did not need tramadol injection anymore. His leukocyte, CRP, PCT declined to 12.00 × 10^9^/L, 66.12 mg/mL, 0.184 ng/mL, and the blood culture was negative. Antibiotic treatment was switched to amoxicillin/clavulanate potassium 0.375g q.8.h and levofloxacin 0.5g q.d oral administration when discharge. In the first month follow-up, the patient reported the fever and low back pain did not recurrent or aggravated, which permit him to stand and walk slowly. We seriously suggested the patient to strictly follow our orders of oral medicine treatment and return visit.

## 3. Discussion

*C fetus* is a slender, curved microaerophilic gram-negative bacterium inhabiting commensally in the intestines of livestock, such as cattle and sheep, and the newly reported reptile: turtle.^[6,7]^ It probably infected human through intake of contaminated food or water. Invasive *C fetus* has been reported to cause pericarditis,^[8]^ endocarditis,^[9]^ pneumonia with empyema,^[10]^ septic arthritis^[11]^ and septic thrombophlebitis.^[12]^ Likewise, Cypierre et al^[13]^ reported the secondary *C fetus* septic sites in lung, joint, skin, periton, and renal. It was believed that, *C fetus* more often caused bacteremia in elder patients (mean aged 68.4^[14]^) with immunosuppression or underlying diseases.^[15]^ The 66-year-old patient in our case admitted that he had contacted livestock when he was a slaughter, and nowadays he has raised many turtles in his residence. The *C fetus* from the reservoirs probably infected his gastrointestine asymptomatically. Then, owning to the gouty arthritis, the patient has taken plenty of metacortandracin as analgetic, which suppressed the immunity leading to asymptomatic bacteremia and varied systematic complications, such as spondylodiscitis. Within the 12 *C fetus*-induced spondylodiscitis cases,^[16]^ only 2 reported secondary psoas abscess which were similar to our case^[3,4]^ (Table 1). Compared with these 2 cases, only psoas was infected without lumbar and intervertebral discs involvement in our case. We checked the patient’s lumbar and intervertebral discs in the MRI, there was no spondylitis sign including irregular boundary, erosion or abscesses (low signal intensity in T1 and high signal intensity in T2), and the fracture lines of the compressive T12 and L1 were clear. The MRI was considered the most superior imaging modalities for early diagnosis of spondylitis with advantages of high sensitivity (96%) and specificity (92%–94%).^[17]^ However, it was reported that whether MRI shows spondylitis signal may depend on the phase of disease.^[18,19]^ Tanaka et al^[19]^ reported MRI did not showed spondylitis signal in 13 days after onset of fever and low back pain, but showed in 26 days after. The patient in our case recalled his low back pain started several years ago, then significantly aggravated in 1 month. Hence, his MRI should have shown spondylitis sign if his lumbar and intervertebral discs were infected. Yet, our radiologists and orthopedists did not agree with any spondylitis in the MRI. Meanwhile we noticed the abnormal tenderness combined with “unknown suborbicular lesions” described in MRI report in the patient’s psoas, and subsequently performed ultrasound-guided puncture biopsy followed with pathogen NGS to indicate the *C fetus*-induced abscess in the psoas. However, it was unclear how the *C fetus* merely infected the psoas but not the lumbar or intervertebral discs. The reason may be that, the patient recalled he had received multiple local injection, probably contained steroids, in the psoas to resolve the severe low back pain, which damaged the immunological barrier of psoas. Within the abscess, pathological examination revealed eosinophils which was probably related to *C fetus* infection, for *Campylobacter* were indicated associating with neutrophilic to eosinophilic switches,^[20]^ and contributing mostly to the eosinophil-associated antimicrobial resistance genes.^[21]^ These hinted the difficulty in treatment toward the *C fetus*-induced primary psoas abscess in our case.

**Table 1:** Case reports of *Campylobacter fetus* spondylitis combined with psoitis.

Date	Author	Age	Sex	Symptom	Comorbidity	Positive culture	Treatment	Outcome
2018	Laenens et al	53	Male	Low back pain	HIV	Intervertebral disc	Oral ciprofloxacin 0.5 g b.i.d	Recovery
2010	Chaillon et al	91	Female	Fever, lower back pain	None	Intervertebral disc	Oral amoxicillin, 6 g q.d	Recovery

With regard to treatment and prognosis, even if the 2 reported cases had full recovery,^[3,4]^ up to 15% fatality rate was reported in patient with *Campylobacter* infection receiving inappropriate antibiotic treatment.^[13,22]^ Due to the rare case, the consensus of *C fetus* infection treatment has not been established till present. As blood cultures were negative for 3 times, which was accordance with the low positive rate reported previously,^[23]^no susceptibility testing could be performed. On initiation, empiric ceftriaxone and levofloxacin i.v. treatment was performed according to the consultation of infectious disease specialist. The patient appeared acute gouty arthritis as previous, with fever and elevated WBC, CRP and PCT, but the puncture induced bacteremia could not be ruled out. Besides, the risk of antibiotic resistance could not be ignored, for *C fetus* was probably resistant to the third generation cephalosporins and fluoroquinolones^[24]^; additionally the eosinophils closely related with antibiotic resistance of *C fetus*^[21]^ were observed in the abscess. Thus, we escalated the antibiotic treatment to meropenem, and the clinical outcome was favorable.

## 4. Conclusion

To sum up, we reported the first case of *C fetus*-induced primary psoas abscess in human worldwide to the best of our knowledge. Although *C fetus* infection was still rare, physicians should notice any patient who had contacted with reservoirs and suffered from immunosuppression and underlying diseases. Diagnosis could be indicated according to the NGS, or other pathogen gene sequencing examination, of the lesion if the culture was negative. Pathological examination is necessary. If the eosinophils were observed, the *C fetus* may have antibiotic resistance, and we recommend the carbapenem antibiotic as the first-line treatment.

## Acknowledgments

The authors would like to thank all additional people, including nurses and specialists for consultation, involved in the patient care at department of general practice.

## Author contributions

**Conceptualization:** Yuan Sijie.

**Data curation:** Xiaodong Luo, Daogang Zha, Yuan Sijie.

**Formal analysis:** Yanfang He, Chunyu Kang, Yuan Sijie.
